# Educational Experiences Within the Learning Community: ECHO Effects on Participants and Clients

**DOI:** 10.1093/geroni/igab046.1677

**Published:** 2021-12-17

**Authors:** Yumi Shirai, Kathleen Bishop

**Affiliations:** 1 University of Arizona, Tucson, Arizona, United States; 2 National Task Group on ID and Dementia Practices, Lee Center, New York, United States

## Abstract

Although Project ECHO is a well-established, effective model to promote quality care in the general healthcare field, this project is the first attempt to implement the model with community care providers of individuals with IDD who are affected by dementia. In order to capture spokes’ (community providers and NTG-Affiliated Regional Trainers) experiences and explore potential benefits, we conducted a content analysis of qualitative data gathered from a spoke feedback session and follow-up surveys from the same group (n = 20). Our findings indicated that spokes appreciated (1) didactic sessions by gaining new knowledge; (2) in-depth, mutual discussion about their cases; (3) experts taking time and ensuring their good practices; and (4) the ability to apply this gained knowledge to their day-to-day practices to improve quality of life for individuals with IDD and their families. We will discuss specific examples that inform future practices.

